# Assessing the convergent validity between the automated emotion recognition software Noldus FaceReader 7 and Facial Action Coding System Scoring

**DOI:** 10.1371/journal.pone.0223905

**Published:** 2019-10-17

**Authors:** Tanja Skiendziel, Andreas G. Rösch, Oliver C. Schultheiss

**Affiliations:** Department of Psychology, Friedrich-Alexander University, Erlangen, Germany; Ghent University, BELGIUM

## Abstract

This study validates automated emotion and action unit (AU) coding applying FaceReader 7 to a dataset of standardized facial expressions of six basic emotions (Standardized and Motivated Facial Expressions of Emotion). Percentages of correctly and falsely classified expressions are reported. The validity of coding AUs is provided by correlations between the automated analysis and manual Facial Action Coding System (FACS) scoring for 20 AUs. On average 80% of the emotional facial expressions are correctly classified. The overall validity of coding AUs is moderate with the highest validity indicators for AUs 1, 5, 9, 17 and 27. These results are compared to the performance of FaceReader 6 in previous research, with our results yielding comparable validity coefficients. Practical implications and limitations of the automated method are discussed.

## Introduction

An emotion is considered an affective reaction to a stimulus that leads to temporary changes of experience and behavior, specifically in the domains of subjective experience, cognition, physiology, motivation and expression [1]. Depending on the specific research question, different methods are employed to measure specific aspects of emotions. For the domain of emotional expression, facial signals represent a central source of information [2], [3]. To tap into these signals, observer-based methods of facial expression measurement have been developed, with the Facial Action Coding System (FACS) by Ekman, Friesen, and Hager [4] representing one of the most frequently used and influential methods. This coding technique enables the objective description of facial expressions based on individual muscle activations. Although this method allows reliable and comprehensive coding of expressions and emotions [2] it is also very labor-intensive [5]. The coding of one minute of video material can take up to two hours depending on the desired accuracy [6]. Furthermore, approximately 100 hours of training are necessary to achieve FACS certification as a coder [7]. Thus, FACS coding is rarely conducted in larger samples, particularly when it involves extended video recordings, and thus a vast number of frames to code, for each target person. By now, automated facial coding software, such as FaceReader (FR) [8], has been developed and could become an alternative to traditional manual coding as these new techniques specifically address disadvantages of previous facial coding methods. The crucial feature of such software that remains to be examined is its validity and convergence with the well-validated manual FACS coding method. FR, in particular, aims to capture through automated algorithms what FACS allows coders to do through manual assessment–that is, the classification of basic facial emotions and the nuanced quantitative measurement of specific AU activity. Our study aims to validate FR trough the comparison between manual and automatic AU coding and emotion classification on the same stimulus set.

Theories and research by Ekman indicates that there is a certain number of discrete, basic emotions that can be differentiated based on multiple characteristics [9–12]. A key requirement of basic emotions is that they involve distinctive universal signals. This requirement is fulfilled by specific facial expressions [9–12]. Facial muscles and their activations are the basis for facial expressions. When a muscle is contracted, the tissue covering it is pulled towards the muscle’s basis on the bone and forms a wrinkle in the direction of the contraction [4]. If the muscular activity involves the simultaneous stimulation of several muscles, a unique constellation of changes appears on the face–such as the raised eyebrows, wide-open eyes, and gaping mouth of surprise—that represents a specific facial expression. Ekman [13] postulated a systematic relationship between distinct emotions and activation patters of specific facial muscles. This means that specific patterns of muscular activations in the face can be seen as a criterion for the activation of an emotion [14]. It also implies that facial expressions of emotions can be dissected into their muscular components [13]. If the criterion of a distinctive facial expression is considered for the definition of a specific emotion [14] [12], then six emotions—happiness, sadness, anger, disgust, fear, and surprise—can be differentiated. (Ekman and Cordaro [12] also postulated a seventh basic emotion–contempt—, but this claim is still a matter of debate; e.g., [15]). As these emotions feature distinctive facial expressions, based on specific patterns of muscle activations, an objective assessment of these emotions by manual and automated coding is possible. Furthermore, FR analysis allows the coding of the presence of basic emotion expressions in facial expressions in general.

Coding emotions based on unique patterns of facial muscle activations forms the conceptual foundation for FACS, which has evolved into the leading facial coding technique [2]. FACS separates between the description of muscle activations and the interpretation of their patterns in terms of specific facial expressions of emotions. It also comes with a detailed coding manual and extensive validation, comprising many studies documenting its reliability and validity (see, for instance, [7, 16]) and iterative refinement of the coding system [4, 17]. These features set it apart from other coding systems, as extensively discussed by Cohn and Ekman [7]. To code all potential facial expressions, FACS targets 44 action units (AUs) based on distinctive muscle activations [2] (see [4] for an overview). The stronger the AU activation, the bolder or more distinct the respective change on the facial surface becomes. FACS scores this intensity on a six-point scale (0 = none, 1 = trace, 2 = slight, 3 = marked/ pronounced, 4 = severe/ extreme, 5 = maximum). The classification of emotions through facial expressions is based on specific patterns of AU activations that are described in the FACS manual [4]. Reliability of FACS coding is usually computed as interrater reliability. The criterion to reach FACS certification is ≥ .70 congruence with expert coding [18]. FACS coding reliability for new material depends on its quality and features as well as the desired extent of coding. Coding reliability is usually high (Κappa > .60), even for spontaneous expressions and the assessment of individual AUs [2], [16]. Validity of FACS coding is often assessed via the criterion of instructed facial expressions and subsequent FACS codings of these expressions [2].

A different method for assessing the validity of FACS is the comparison between manual and automatic AU coding with specific software [2]. The inversion of this approach, validating a facial-coding software via FACS coding of instructed facial expressions of basic emotions, is the central objective of our study.

Automated facial coding could offer an attractive alternative to classic FACS coding as it drastically decreases the time needed for both learning and applying the method. Some researchers (e.g., [5] [7]) even consider the former to have greater objectivity and reliability than the latter, because repeated analyses of the same material will always yield exactly the same result in the case of automated coding. Subjective coding bias and fatigue effects are completely absent. Thus, a well validated automated coding method has the potential to extend emotion research by making analysis of facial expressions more accessible and affordable.

Automated facial coding methods can be distinguished according to two features. The first is the distinction between commercially available software and approaches specifically developed for individual studies. While commercial software is typically used to examine hypotheses, but their validity is only claimed by the manufacturer and cannot be examined by looking at the software code, custom-tailored approaches offer insight into the technical background and reliability and validity of automated coding [5], [7]. The second important distinction is the one between descriptive and interpretive coding approaches. Descriptive techniques offer the segmentation of facial expressions into their specific components such as AUs, without necessarily delivering a classification of the emotion a specific combination of components points to (see [5], [19], [20]), while interpretive approaches only allow the holistic classification of emotions, based on global detection algorithms whose exact operational characteristics are usually not made transparent in the software’s documentation or output.

A comprehensive professional software for facial coding is Noldus FR version 7 [8]. As this software will be used in this study we will briefly describe and compare it to other similar programs such as Affdex [21], EmoVu [22], and nViso [23]. FR was developed and validated on the basis of psychological emotion theories, specifically Ekman’s research and the FACS. The software allows the coding of the emotions happiness, sadness, anger, disgust, fear, surprise and contempt as well as a neutral expression. An additional AU module also enables the coding of 20 FACS AUs (AUs 1, 2, 4, 5, 6, 7, 9, 10, 12, 14, 15, 17, 18, 20, 23, 24, 25, 26, 27 and 43; for illustrations of these AUs, please see, for instance, [4]). This module focuses on the most frequent and relevant AUs, leaving out AUs related, for instance, to head turn and tilt. AU activation intensity is measured as continuous values from zero (no activation at all) to one (maximum activation), in increments capturing intermediate stages labeled “trace”, “slight”, “pronounced”, and “severe”, mirroring the classification of AUs in FACS and corresponding to specific intervals on the zero-to-one scale [8]. Although the manual does not explain the algorithm involved in AU measurement in any detail, it describes the process of classifying basic facial emotions as consisting of three steps: 1) detection and localization of a face; 2) parallel analysis through (a) a classifier based on 500 key points in the face and trained with more than 1000 manually coded facial expressions and (b) a deep artificial neural network for pattern recognition; (3) integration of the results of the parallel analysis processes into an emotion classification [8].

Other examples for professional facial coding software are Affdex [21], EmoVu [22], and nViso [23]. These methods differ from FR in two central aspects. Firstly, they only offer the interpretive feature of overall emotion classification and scaling, not the coding of single AUs (note however, that Affdex, after it was integrated into iMotions, now also provides analysis of 20 AUs–see https://imotions.com/biosensor/fea-facial-expression-analysis/). Secondly, these approaches are live or online tools, directed at marketing research and research on consumer behavior, but do not allow the analysis of still pictures or previously recorded material. Thus the choice of the appropriate automated software depends on the specific research question and material.

As automated facial coding methods might be an economic alternative to manual FACS coding, the assessment of their validity and the comparison with FACS psychometrics, the de facto gold standard of facial expression assessment [2], becomes increasingly important. If automated coding is similar to manual coding, particularly when applied to instructed facial expressions of emotion, then convergent validity of the automated method with the established manual method can be tested [7]. Correlations between the manual and automated coding of individual AUs as well as indices of precision, recall, and accuracy can thus be used as measures of validity [6] [24]. Percentage agreement can be used to assess the convergence between automated emotion classifications and the criterion of instructed facial expressions of emotion.

Overall there is supportive evidence for the validity of automated facial coding. Bartlett et al. [5] report 91% congruence with instructed-AU expressions. The expert FACS coding of the same dataset reached 92% congruence with the instructed pattern and does not differ significantly from the automated coding. Similarly high indicators were found by Tian et al. [20]. Here an automated coding method identified 93% of AU activations (limited to 16 AUs) coded by FACS on two datasets. Automated methods even yield results equivalent to manual coding when tested on stimulus sets of spontaneous expressions, as a study by Kapoor et al. [19] shows. The congruence between automated and FACS coding reached 69%, which is an appropriate score regarding the differences between spontaneous and instructed facial expressions, such as lower intensities of activations, covering of facial areas or non-frontal positioning of the face that make the scoring of spontaneous material generally more demanding [2].

There is also some specific validity evidence for FR. Bijlstra and Dotsch [25] examined the emotion classification of FR (version 4) vis-à-vis prototypical emotion enactment according to FACS and report 90% correct classifications for their dataset. Rates of correct classification varied depending on the target emotion, with the highest accuracy for happiness (96%) and surprise (94%). The lowest accuracy emerged for fear and disgust (85% each). For version 6 of FR, Lewinski et al [6] report an overall agreement index (F1, an integration of measures of precision and recall, see below) of .67 for two datasets that were coded for AU intensities by two independent FACS coders. The authors classify this result as acceptable, as it is rather close to the criterion of .70 agreement with expert coding necessary to reach FACS certification. In this study, the emotion classification of FR was also tested. The averaged congruence with the intended emotion categories reached 88% in the two datasets. Similar to the findings of Bijlstra and Dotsch [25], correct classification varied depending on the target emotion, with the highest averaged accuracy score for happiness (96%), followed by surprise and neutral (94% respectively), disgust (92%), sadness (86%), fear (82%) and lastly anger (76%).

Although version 7 of FR has already been used in multiple research fields (e.g. consumer behavior: [26]; pain research: [27]), validation of the software is sparse. The purpose of our study is to contribute to the general validation of FR 7 by assessing the convergent validity with FACS coding on an independent dataset. Both the emotion classification and the AU coding features of FR 7 are examined for their congruence with instructed emotion expressions and FACS codings, respectively. More specifically, we determined the portion of correct emotion classifications for every emotion, using percentage values. Furthermore, we examined if the FR’s option to calibrate the assessment of emotional-expression stimuli based on a comparison with corresponding neutral expressions contributes to improved classification results by comparing percentage values of correct classifications on the same dataset without and with calibration. Concerning the AU module of FR 7, we investigated the congruence between automated and FACS-based intensity coding using correlations. We expected significant positive correlations between the manual and automatic coding for individual AUs constituting specific emotional expressions according to the FACS manual. For instance, we expected AUs 6 and 12, whose combined activation is critical for classifying an emotional expression as happiness [4], to show good convergent correlations (i.e., ≥ .60, [28]) between FACS and FR.

## Method

### The standardized and motivated facial expression of emotion dataset

The original acquisition of the video material used in this research was approved by Friedrich-Alexander University's legal review department, which, at the time the material was recorded, constituted the equivalent of an Institutional Review Board or Research Ethics Committee for the behavioral sciences that independently evaluated human subject research ethics. Informed consent was obtained from all research participants. Participants were treated in accordance with the ethical principles outlined in the 1964 declaration of Helsinki.

Our study was based on the *Standardized and Motivated Facial Expression of Emotion* (SMoFEE) stimulus set [29]. The dataset contains static (pictures) and dynamic (movies) facial expressions enacted by 80 Caucasian individuals (36 men; *M* = 22.63 years, *SD* = 2.43). Each individual enacted in a prototypical fashion patterned after the *Japanese and Caucasian Facial Expressions of Emotion (JACFEE) and Neutral Faces (JACNeuF)* slide set [30] the emotions happiness, sadness, anger, disgust, fear and surprise as well as two neutral expressions (mouth open and closed). In addition, they also freely enacted each emotion for motivational contexts related to power, achievement and affiliation, as prompted by prerecorded narrative vignettes. In total, each individual encoded 25 expressions, resulting in 2,000 videos and stills. Because several participants who enacted the emotions indicated on their informed consent form that they do not want their pictures and movie clips to be publicly available through the internet, we cannot provide a link to the stimulus set. However, provided that potential users agree in writing to honor this stipulation, the SMoFEE stimulus set can be obtained upon request from the second or third author. For examples of the picture set, please see the supplementary materials posted on the Open Science Framework (https://osf.io/zpjqy/). For all pictures, FACS codings of the intensities of AU activations and classifications of the prototypicality of the emotion expressions are included. For more information on the coding and validation of the SMoFEE stimulus set, please see https://opus4.kobv.de/opus4-fau/files/2304/AndreasRoeschDissertation.pdf [29]. In the following, we will focus only on the subset of photographs showing standardized emotional expressions, because these represent a common validity standard in research on facial emotions and allow direct comparisons with other studies employing pictures of prototypical emotions [29–31].

#### Equipment

A SONY HDR-SR8E video camera with HD 1440 x 1080 resolution was used for video recordings of the emotion expressions. To optimize the lighting conditions, two dimmable panel lights with 110 W each and a blue background screen were used. During the shots, participants sat on a chair, with the camera positioned 160 cm in front of the chair and the two panel lights positioned 40 cm to the right and left of the camera. The blue screen was positioned 140 cm behind the subject.

#### Procedure

The recording of the standardized expressions took 60 minutes on average. To achieve a high level of standardization, participants were asked to take off their jewelry and to move their hair out of the face. A black hairdressing cape was used to cover visible clothing. For the depiction of the basic emotions participants were asked to mimic the respective expression of JACFEE reference pictures as accurately and naturally as possible. One of the two experimenters present during the recording of the videos was a certified FACS trainer; the other experimenter had been instructed by the first experimenter. Participants were given a mirror to examine and practice their mimic. For the emotion depiction during the video shot, participants were instructed to start with a neutral expression, then perform the emotion expression and to end with a neutral expression again. Each emotion condition was repeated at least twice, with the experimenter first providing performance instructions for the encoding of the emotion, then controlling the first encoding and providing feedback to help participants improve their performance. The subsequent second encoding was recorded. If an expression still failed to satisfactorily replicate the JACFEE template, participants were asked to repeat encoding the expression until a satisfactory result was achieved.

#### Data processing and FACS coding

The recording sessions used in present research yielded a total data pool of 640 (80 participants x 8 conditions, resulting from 6 emotions, one neutral condition with mouth open and one neutral condition with mouth closed) standardized video sequences. The software *Picture Motion Browser* by SONY was employed to view the videos. Subsequent cutting, editing, and the creation of still images was carried out using the software Adobe Premiere 3.0. Microsoft Windows Photo Gallery was used to view the stills.

#### Creating coding templates

For every recorded video sequence, both a static and dynamic coding template of the maximal emotion expression were created for subsequent FACS coding. The first step was selecting the video sequence with the maximal expression intensity. Next, clips were cut following predefined criteria for the beginning and end of an emotion. These clips thus started with brief depiction of an initially neutral expression and then showed the waxing and waning of the emotional expression itself. In a third step, still pictures were created by identifying the frame depicting the maximal emotion expression. The person editing the videos was trained in FACS coding and determined the maximal expression according to expression intensity and prototypicality according to the FACS manual.

#### FACS coding

All 640 still images were FACS coded for the intensity of the activated AUs on a six-point scale (0 = none, 1 = trace, 2 = slight, 3 = marked, 4 = severe, 5 = maximum). Two coders certified for FACS each coded half of the pictures. Fifty stills were double-coded by both coders for reliability determination. Interrater reliability of coding was .83 (agreement index), exceeding the criterion for FACS certification (> .70) and indicating good reliability. For the coding of an emotion expression the participant’s neutral expression and the respective dynamic emotion sequence were used for reference. Coding of a single expression took 10–15 min and 80–120 min for all 8 expressions of a participant and thus about 13.3 h (100 min x 80 participants = 800 min) for the entire picture pool.

### FR 7 analysis

FR 7 (henceforth simply termed FR) analyses was carried out on a Dell Latitude E5470 Laptop which met the system requirements specified in the FR manual [8]. The picture requirements for FR, such as the minimal image resolution of 640 x 480 pixel, the frontal, close–up, and complete display of the face, as well as even frontal illumination, were met by the SMoFEE pictures. Picture resolution of 1440 x 1080 even exceeded the requirements.

#### Video preparation

The SMoFEE video clips of the standardized emotion expressions happiness, sadness, anger, disgust, fear and surprise as well as the neutral expressions with mouth closed were chosen as source material for FR analysis. Prior to the analysis the video clips had to be converted for further processing by FR. For this step the software *PlayMemories Home* by SONY was used. The target data format was mp4 with 1920 x 1080 resolution and 25 Frames per second. The final dataset thus consisted of 480 emotion sequences (80 participants x 6 emotions) and 80 recordings of the closed-mouth neutral expressions.

#### Video analysis

FR video analysis was conducted in two runs. First, we analyzed videos *without* prior calibration of participants’ neutral expressions, and then we analyzed the same videos with prior calibration. The neutral expression was not analyzed, but used for the calibration.

#### FR settings

The basic FR settings [8] were modified for the analysis. Automatic continuous calibration and frame-to-frame smoothing of classification values were deactivated to ensure high-accuracy raw data and also because we made the calibration against a neutral face an explicit, separate feature of our analysis (see above). The general default face model, which had been trained on a wide variety of images and according to the handbook will work best for most people [8] was selected because it fit SMoFEE’s Caucasian adult participants best. Sample rate was set to every frame. The optional classification of the *contempt* expression was excluded from analysis as it was not featured in the SMoFEE dataset. AU classification was activated. Data export was set to continuous values to ensure full use of all FR output.

#### Data analysis without calibration

The FR *Project Wizard* was used to feed all participants’ video clips, with six emotions per participant, into FR analysis. All videos were automatically and successively analyzed utilizing the batch analysis feature which allowed us to process all videos in one run. This took approximately 90 min.

#### Data analysis with calibration

FR’s calibration feature allows to control for confounding effects of a recoded person’s physiognomy or habitual facial expression in the evaluation of dynamic emotional expressions. Thus, if a neutral expression features aspects of an emotion expression, FR’s emotion coding algorithms can be biased when a neutral or when an emotional expression is presented by that person. To minimize this bias, FR allows to conduct a person-specific calibration based on the features of a neutral expression target person. The calibration is based on the analysis of two seconds of video of a neutral expression, with the algorithm identifying the image with the lowest model error for calibration (for further details, please see [8]). Subsequent changes in emotional expression detected by FR in emotion-expression videos then represent the deviation from the neutral-expression calibration template. Note, however, that calibration only influences emotion classification in FR, not AU coding.

Calibration per participant was carried out using the respective video of the neutral expression with mouth closed. FR does not allow automatic alignment of a calibration to all videos of the respective participant. Thus, for each participant, we manually set up analysis with the respective calibration. The subsequent batch analysis again took 90 min.

#### Data export

After batch analysis data export was respectively carried out on project basis. Both state logs and detailed logs are available as export formats. In our analyses, we only used detailed logs, which provide detailed frame-by-frame information on emotion intensity and AU activations, with values ranging from 0 to 1.

### Statistical procedure

#### Data preparation

The FACS codings of the standardized emotion expressions from the SMoFEE dataset and the detailed logs from the FR analysis without and with calibration served as basis for statistical analyses. The log files showed data gaps for a few frames when no FR analysis could be accomplished. These frames (< 1%) were excluded from further analysis. Considering the high number of analyzed frames for every recording, these few frames carried no weight.

#### Data analysis

The software SYSTAT 13 was used to run all statistical analyses. Additional significance testing was conducted using SPSS Statistics 24. All source data, a SYSTAT processing and analysis script, an SPSS version of the analysis file, and future updates are available from https://osf.io/zpjqy/.

To extract FR’s dominant emotion classification, the maximal intensity of each of the six possible emotion categorizations (*happy*, *sad*, *angry*, *disgusted*, *scared* and *surprised*) was determined for each clip. The emotion categorization with the highest maximal intensity score represents the dominant emotion classification of the video clip. The intensity scores of the categorization *neutral* were excluded from this analysis, as all videos begin and end with a neutral expression. As no shots with intended neutral expression were analyzed, the exclusion of the *neutral* categorization does not impair the examination of the dominant emotion expression.

In the FACS evaluation, AU coding was carried out using the still of the maximal emotion expression of the respective video. To approach the FR data in a similar way, the maximum in the video for each AU over all frames of the entire video was extracted. This method followed the assumption that the frames in which the maximal AU activations occur according to FR correspond to a certain extent with the manually selected frame of the maximal emotion expression of the FACS analysis. As both coding methods used the maxima of the AU activations as basis of the evaluation, the congruence between the manual and automatic coding of the *intensity* of the AU activations could be examined. Only the 20 AUs that are coded by both FR and FACS were included. As the calibration of data in FR does not influence the coding of AUs, the dataset without prior calibration was used for these calculations.

We computed Spearman rank-order correlations as a measure of congruence between the maximum values of the AU activations from FR, assessed on a continuous scale, and the ordinal FACS scale scores of the SMoFEE dataset. Our use of Spearman correlations was also based on the observation that most of the AU variables in both FR and SMoFEE codings were not normally distributed according to either inspection of histograms and/or Shapiro-Wilk tests for normality (*p* < .05). Moreover, we also calculated indices of accuracy, precision, recall, and the integration of the latter two (F1) to allow direct comparisons between our results and those of a similar analysis for an earlier version of FR and different picture sets [6].

## Results

### Descriptive statistics: Emotion classification without and with calibration

The dominant emotion classification of a video sequence–that is, the emotion for which FR calculated the highest likelihood—was calculated for the dataset without and with calibration. The rate of congruence between the dominant emotion classification and the intended emotion referring to the performance condition reflects the degree of correct emotion categorization of FR. If a different than the intended emotion is classified as dominant emotion expression by FR, the coding is classified as incorrect. The proportion of correct and incorrect codings by FR depending on the emotion condition is shown in Table 1.

**Table 1:** Confusion matrix for the intended emotional expressions and the Face Reader (FR) classifications without and with prior calibration of the software.

	Face Reader Classification					
Intended Expression	Happiness	Sadness	Anger	Surprise	Fear	Disgust
Happiness	100%	0%	0%	0%	0%	0%
	(100%)	(0%)	(0%)	(0%)	(0%)	(0%)
Sadness	3.75%	75%	15%	2.50%	2.50%	1.25%
	(3.75%)	(73.75%)	(13.75%)	(3.75%)	(3.75%)	(1.25%)
Anger	6.25%	7.50%	83.75%	0%	0%	2.50%
	(6.25%)	(5%)	(86.25%)	(0%)	(0%)	(2.50%)
Surprise	2.50%	1.25%	1.25%	87.50%	7.50%	0%
	(2.50%)	(0%)	(1.25%)	(90%)	(6.25%)	(0%)
Fear	2.50%	3.75%	7.50%	32.50%	51.25%	2.50%
	(3.75%)	(1.25%)	(6.25%)	(33.75%)	(52.5%)	(2.50%)
Disgust	17.50%	1.25%	5%	0%	0%	76.25%
	(17.50%)	(0%)	(6.25%)	(0%)	(0%)	(76.25%)

*Note*. The intended facial expressions are represented horizontally, the FR evaluations vertically. The numbers in brackets show the classification with prior calibration. Gray fields indicate correct classifications. The total number of videos per emotion condition is *N* = 80.

Without prior calibration FR reached a 79% mean ratio of correct identification. With calibration the classification was correct in 80% of the cases. Both datasets showed varying degrees of correct categorization depending on the emotion conditions. In both cases expressions of the condition *happiness* were consistently identified correctly. In descending order of the degree of correct classification follow the conditions *surprise*, *anger*, *disgust*, *sadness* and *fear* for both analysis options. Although the correct identification rate for *fear* was substantially above chance (= 16.7%), it was only about 50% in both conditions. This expression was most frequently falsely coded as *surprise*. This occurred in both datasets in about one third of the cases. Additionally, in both datasets around one fifth of the disgust expressions were falsely categorized as *happiness* by FR. Overall, differences between the coding of emotion expressions without and with calibration were only marginal.

### Inferential statistics: Congruence between manual and automatic AU coding

Spearman correlations between the FR and FACS coding of the 20 relevant AUs were calculated for each emotion condition. Additional significance tests were only carried out for the congruent correlations in every emotion condition—meaning the coding of the same AU with the two different methods—as other correlations seemed less relevant for the purpose of this research. Table 2 shows the correlations between the manual and automatic coding of AUs structured by the emotion performance conditions. Table 3 provides the corresponding mean and standard deviation values. Because different AUs are relevant for each expression, we focused on the correlations of these essential AUs. The AU configurations according to the FACS Investigator’s Guide form an appropriate guideline for the relevant AUs in each emotion condition [4]. The essential AUs for the respective expression are thus highlighted in Table 3. The correlation matrix shows various missing values due to the fact that either FR, or FACS, or both methods classified these AUs as inactive in all cases.

**Table 2:** Spearman correlations between the coding of action units (AUs) with the Facial Action Coding System (FACS) and automated FaceReader 7 (FR) coding for all emotion expression conditions.

	Happiness	Sadness	Anger	Surprise	Fear	Disgust
AU 1 Inner Brow Raiser	-.04^ns^	.71**	-.05^ns^	.62**	.70**	.77**
AU 2 Outer Brow Raiser	-.03^ns^	.40**	.	.60**	.58**	.53**
AU 4 Brow Lowerer	.	.43**	.30**	-.03^ns^	.53**	.32**
AU 5 Upper Lid Raiser	.11^ns^	.47**	.34**	.72**	.62**	-.08^ns^
AU 6 Cheek Raiser	.46**	.17^ns^	.28*	.	.23*	.23*
AU 7 Lid Tightener	.22^ns^	.39**	.34*	.25*	.33**	.20^ns^
AU 9 Nose Wrinkler	.	.	.76**	.	.	.48**
AU 10 Upper Lip Raiser	.25*	.14^ns^	.54**	.	.10^ns^	.11^ns^
AU 12 Lip Corner Puller	.08^ns^	.27*	.40**	.19^ns^	.11^ns^	.18^ns^
AU 14 Dimpler	.	.42**	-.11^ns^	.	-.02^ns^	.
AU 15 Lip Corner Depressor	.	.44**	.39**	.	.21^ns^	.16^ns^
AU 17 Chin Raiser	.	.58**	.72**	-.03^ns^	.04^ns^	.49**
AU 18 Lip Pucker	.	-.03^ns^	.13^ns^	.33**	.	.
AU 20 Lip Stretcher	-.03^ns^	.56**	.	.	.50**	.42**
AU 23 Lip Tightener	.	.02^ns^	.09^ns^	-.04^ns^	.	.
AU 24 Lip Pressor	.	-.01^ns^	.32**	.	.	.
AU 25 Lips Part	.23*	.	.25*	.22*	.17^ns^	.44**
AU 26 Jaw Drop	.24*	.	-.10^ns^	.20^ns^	.35**	.26*
AU 27 Mouth Stretch	.	.	.	.62**	-.01^ns^	-.02^ns^
AU 43 Eyes Closed	.	.	-.03^ns^	.	.	.

*Notes*. The relevant AUs for the expression of the respective emotion are marked gray.

^ns^ = not significant.

* *p* < .05

** *p* < .01, uncorrected, two-tailed.

**Table 3:** Mean (standard deviation) values for Facial Action Coding System (FACS) and FaceReader (FR) coding of Action Units (AU).

	Happiness		Sadness		Anger		Surprise		Fear		Disgust	
	FACS	FR	FACS	FR	FACS	FR	FACS	FR	FACS	FR	FACS	FR
AU 1 Inner Brow Raiser	0.03 (0.16)	.03 (.13)	2.76 (1.51)	.52 (.38)	0.03 (0.16)	.06 (.19)	4.25 (1.28)	.69 (.29)	3.38 (1.46)	.59 (.34)	0.26 (0.90)	.05 (.18)
AU 2 Outer Brow Raiser	0.06 (0.33)	.02 (.10)	0.89 (1.48)	.09 (.23)	0.00 (0.00)	.01 (.07)	4.26 (1.25)	.72 (.30)	2.81 (1.69)	.49 (.37)	0.26 (0.94)	.02 (.11)
AU 4 Brow Lowerer	0.00 (0.00)	.02 (.06)	2.69 (1.26)	.52 (.36)	3.88 (1.27)	.69 (.27)	0.03 (0.22)	.02 (.09)	1.81 (1.70)	.28 (.31)	3.49 (1.68)	.48 (.30)
AU 5 Upper Lid Raiser	0.06 (0.29)	.07 (.19)	0.16 (0.56)	.17 (.28)	0.26 (0.90)	.29 (.32)	2.70 (1.75)	.72 (.22)	2.91 (1.52)	.68 (.26)	0.05 (0.27)	.09 (.22)
AU 6 Cheek Raiser	2.28 (1.43)	.76 (.20)	0.29 (0.93)	.02 (.10)	0.29 (0.93)	.06 (.17)	0.00 (0.00)	.02 (.08)	0.08 (0.38)	.03 (.14)	1.49 (1.84)	.44 (.34)
AU 7 Lid Tightener	2.14 (1.44)	.19 (.27)	1.19 (1.46)	.27 (.33)	2.88 (1.66)	.53 (.23)	0.03 (0.22)	.06 (.16)	0.20 (0.64)	.10 (.22)	3.88 (1.26)	.71 (.20)
AU 9 Nose Wrinkler	0.04 (0.25)	.00 (.00)	0.00 (0.00)	.01 (.07)	0.40 (1.20)	.06 (.20)	0.00 (0.00)	.00 (.00)	0.05 (0.35)	.00 (.00)	4.01 (1.35)	.56 (.34)
AU 10 Upper Lip Raiser	0.04 (0.34)	.16 (.28)	0.10 (0.38)	.04 (.16)	0.20 (0.74)	.06 (.20)	0.00 (0.00)	.02 (.12)	0.21 (0.63)	.08 (.22)	2.91 (1.68)	.70 (.26)
AU 14 Dimpler	0.00 (0.00)	.05 (.17)	0.09 (0.46)	.06 (.20)	0.64 (1.31)	.08 (.20)	0.00 (0.00)	.02 (.10)	0.03 (0.22)	.02 (.12)	0.00 (0.00)	.02 (.10)
AU 15 Lip Corner Depressor	0.00 (0.00)	.05 (.18)	0.91 (1.40)	.42 (.39)	0.13 (0.68)	.12 (.27)	0.00 (0.00)	.02 (.09)	0.08 (0.47)	.09 (.24)	0.16 (0.74)	.14 (.28)
AU 17 Chin Raiser	0.00 (0.00)	.07 (.19)	1.75 (1.60)	.42 (.39)	2.73 (2.08)	.50 (.35)	0.03 (0.22)	.03 (.14)	0.15 (0.51)	.06 (.19)	0.59 (1.45)	.13 (.25)
AU 18 Lip Pucker	0.00 (0.00)	.01 (.07)	0.03 (0.22)	.05 (.18)	0.30 (1.02)	.08 (.21)	0.03 (0.22)	.05 (.16)	0.00 (0.00)	.06 (.17)	0.00 (0.00)	.02 (.10)
AU 20 Lip Stretcher	0.03 (0.22)	.03 (.13)	0.11 (0.60)	.04 (.17)	0.00 (0.00)	.02 (.12)	0.00 (0.00)	.05 (.16)	0.46 (1.23)	.33 (.40)	0.46 (1.22)	.12 (.26)
AU 23 Lip Tightener	0.00 (0.00)	.04 (.15)	0.21 (0.84)	.19 (.30)	1.39 (1.91)	.74 (.25)	0.01 (0.11)	.08 (.22)	0.00 (0.00)	.11 (.26)	0.00 (0.00)	.15 (.29)
AU 24 Lip Pressor	0.00 (0.00)	.11 (.26)	0.10 (0.44)	.28 (.35)	2.56 (1.81)	.74 (.27)	0.00 (0.00)	.14 (.27)	0.00 (0.00)	.12 (.26)	0.00 (0.00)	.17 (.29)
AU 25 Lips Part	3.58 (0.84)	.09 (.08)	0.00 (0.00)	.04 (.13)	0.05 (0.45)	.10 (.25)	2.99 (0.41)	.77 (.17)	3.00 (0.62)	.70 (.19)	3.75 (0.92)	.80 (.17)
AU 26 Jaw Drop	1.21 (1.22)	.10 (.17)	0.05 (0.27)	.00 (.00)	0.25 (0.79)	.04 (.14)	2.96 (1.91)	.55 (.25)	2.10 (1.35)	.30 (.29)	1.58 (1.39)	.17 (.21)
AU 27 Mouth Stretch	0.00 (0.00)	.00 (.00)	0.00 (0.00)	.00 (.00)	0.00 (0.00)	00 (.00)	0.60 (1.24)	.19 (.31)	0.03 (0.22)	.01 (.07)	0.05 (0.31)	.01 (.07)
AU 43 Eyes Closed	0.00 (0.00)	.66 (.39)	0.00 (0.00)	.73 (.35)	0.03 (0.22)	.52 (.41)	0.00 (0.00)	.65 (.41)	0.00 (0.00)	.71 (.37)	0.00 (0.00)	.56 (.42)
AU 12 Lip Corner Puller	4.04 (0.91)	.90 (.07)	0.06 (0.29)	.04 (.13)	0.06 (0.33)	.15 (.26)	0.06 (0.29)	.11 (.22)	0.04 (0.25)	.12 (.22)	0.11 (0.50)	.33 (.30)

*Note*. The relevant AUs for the expression of the respective emotion are marked gray. Range for FACS values: 0–5. Range for FR values: 0–1.

Finally, Table 4 presents additional evaluation metrics for assessing the performance of AU measurement in FR. *Presence* refers to the frequency with which FR and FACS coding detected an AU activation in the 480 emotion sequences. *Recall* gives the ratio of FACS-coded AUs detected by FR (i.e., FR/FACS). Precision is a ratio of how often FaceReader is correct when classifying an AU as present (i.e, FACS/FR). *F1* summarizes the trade-off between recall and precision via the formula: 2 x [(Precision x Recall)/(Precision + Recall)]. *Accuracy* gives the percentage of correct classification according to the formula: (correctly classified AU absence + correctly classified AU presence)/number of emotion clips. For more information on these indices, please see [6]. The results presented in Table 4 indicate that FR detected AUs more frequently in the full emotion clips than FACS detected them in the maximal-expression still images. Results for the F1 measure, which best reflects the FACS category agreement calculation (see [6]), suggest that FR measurements of AUs 1, 2, 4, 5, 6, 7, 9, 10, 12, 17, and 25 all exceeded the .70 threshold needed to pass the FACS calibration test and therefore represent sufficiently precise assessments of the activation of these AUs. FR measurements of AUs 24, 26, and 27 performed in the acceptable range (.60 to .70), and FR measurements of AUs 14, 15, 18, 20, 23, and 43 performed poorly (< .60). In general, the more frequently an AU activation occurred in the picture and film material, the better FR performed. Conversely, the AUs for which FR showed the poorest performance were also those that were infrequent either in the maximum-emotion still images (i.e., AUs 18, 20, 23 and 43) or in both the still images and the full clips (i.e., AUs 14 and 15).

**Table 4:** FaceReader (FR) and Facial Action Coding System (FACS) coding frequencies and FaceReader performance for five evaluation metrics of action unit (AU) classification.

	Present		Recall	Precision	F1	Accuracy
Action Unit	FACS	FR				
AU 1 Inner Brow Raiser	230	207	0.82	0.94	0.88	0.88
AU 2 Outer Brow Raiser	178	141	0.75	0.95	0.84	0.88
AU 4 Brow Lowerer	265	248	0.83	0.90	0.86	0.84
AU 5 Upper Lid Raiser	163	229	0.92	0.69	0.79	0.80
AU 6 Cheek Raiser	123	154	0.85	0.67	0.75	0.84
AU 7 Lid Tightener	251	249	0.76	0.81	0.78	0.75
AU 9 Nose Wrinkler	89	70	0.79	0.97	0.87	0.95
AU 10 Upper Lip Raiser	88	121	0.86	0.69	0.77	0.86
AU 12 Lip Corner Puller	96	202	0.97	0.57	0.72	0.76
AU 14 Dimpler	21	31	0.13	0.09	0.11	0.90
AU 15 Lip Corner Depressor	38	99	0.69	0.37	0.48	0.82
AU 17 Chin Raiser	127	143	0.76	0.71	0.73	0.84
AU 18 Lip Pucker	9	38	0.38	0.13	0.19	0.91
AU 20 Lip Stretcher	26	66	0.69	0.28	0.40	0.89
AU 23 Lip Tightener	36	151	0.81	0.28	0.42	0.73
AU 24 Lip Pressor	64	170	0.97	0.51	0.67	0.76
AU 25 Lips Part	317	341	1.00	0.94	0.97	0.95
AU 26 Jaw Drop	235	184	0.60	0.76	0.67	0.69
AU 27 Mouth Stretch	20	25	0.69	0.54	0.61	0.96
AU 43 Eyes Closed	1	355	1.00	0.00	0.00	0.26
*Average*	*118*.*85*	*161*.*20*	*0*.*76*	*0*.*59*	*0*.*63*	*0*.*81*

#### Happiness

AUs 6 (cheek raiser) and 12 (lip corner puller, *zygomaticus major*) are responsible for this expression, but a significant positive correlation between the manual and automatic coding could only be confirmed for AU 6—for AU 12 we found no substantial correlation despite good between-measure agreement (F1), perhaps due to a ceiling effect leading to restricted variance in FR and FACS measurements (see Table 3) and hence also restricted covariance between them. Additional significant correlations were observed for AUs 10, 25 and 26.

#### Sadness

For the expression of sadness, AUs 1 (inner brow raiser), 4 (brow lowerer, *corrugator supercilii*), 6 (cheek raiser), 15 (lip corner depressor), 25 (lips part) and 26 (jaw drop) are relevant. As expected, we found positive correlations for AUs 1, 4 and 15. For AU 6 the correlation did not reach significance, while for AUs 25 and 26 no substantial correlations emerged. Additional significant connections were found for AUs 2, 5, 7, 12, 14, 17 and 20.

#### Anger

The activation of AUs 4 (brow lowerer), 5 (upper lid raiser), 7 (lid tightener), 10 (upper lip raiser), 17 (chin raiser), 23 (lip tightener), 24 (lip pressor), 25 (lips part) and 26 (jaw drop) is associated with the facial expression of anger. We observed congruence between the manual and automatic coding of AUs for AUs 4, 5, 7, 10, 17, 24 and 25. Only for AUs 23 and 26 no significant correlations occurred. These AUs were also characterized by unsatisfactory performance indices (i.e, F1). Further connections appeared for AUs 6, 9, 12 and 15.

#### Surprise

This expression is characterized by activations of AUs 1 (inner brow raiser), 2 (outer brow raiser), 5 (upper lid raiser), 25 (lips part), 26 (jaw drop) and 27 (mouth stretch). For five out of the six relevant AUs (1, 2, 5, 25, and 17) we found significant convergence between FACS and FR. For AU 26 no significant result emerged. Consistent with the lack of convergence in our correlation analyses, AUs 26 and 27 performed poorly overall according to the F1 index of agreement (see Table 4). For AUs 7 and 18 manual and automatic coding converged, too.

#### Fear

The characteristic expression of this emotion recruits AUs 1 (inner brow raiser), 2 (outer brow raiser), 4 (brow lowerer), 5 (upper lid raiser), 20 (lip stretcher), 25 (lips part), 26 (jaw drop) and 27 (mouth stretch). We detected significant correlations for AUs 1, 2, 4, 5, 20 and 26, but not for AUs 25 (despite excellent overall agreement according to the F1 index) and 27. Additional significant connections occurred for AUs 6 and 7.

#### Disgust

The expression of disgust can be traced to activation of AUs 9 (nose wrinkler), 10 (upper lip raiser), 15 (lip corner depressor), 17 (chin raiser), 25 (lips part) and 26 (jaw drop). We found congruence between the FR and FACS coding for AUs 9, 17, 25 and 26. For the coding of AUs 10 and 15 we observed no significant correlations. The latter AU was also characterized by poor between-measure agreement overall according to the F1 index. Additional significant positive correlations emerged for AUs 1, 2, 4, 6 and 20.

## Discussion

Although FACS represents a pioneering method of emotion research, the coding system is very time-consuming, and this might partially impede its actual usage. Automated facial coding software largely eliminates this disadvantage by offering the promise of valid emotion classification and AU coding for a fraction of the usual time. The objective of this study was to verify the validity of such automated methods using FR version 7 [8]. The convergence between manual and automated coding of AU intensities and the degree of correct emotion classification were determined. Overall the results of this study support the validity of automated coding methods.

### Accuracy of emotion coding

FR accomplished correct classifications of the intended emotion expressions in 79% and 80% of the cases without and with calibration, respectively. These and all following classification rates need to be compared to a baseline of 16.7% representing chance performance. Happy expressions were consistently coded correctly. For the other emotions FR always identified the intended expression with the highest likelihood; however incorrect classifications occurred, too. Expressions of surprise and anger had the lowest rates of false codings. In contrast, almost half of the fear expressions were classified incorrectly by the software. Notably, however, even in this case FR did not fare substantially worse than human coders tested across many studies (i.e., 39% to 90%) [32], possibly due to the high similarity between AU activation patterns of fear and surprise [33]. For all other expressions FR performed consistently better than the average human coder [32]. We conclude from our findings that for the emotions happiness, surprise, anger, disgust and sadness, FR-based categorization can be rated as valid, while the identification rate for fear constitutes a limitation for FR’s capacity that is similar to average human coders’ [32].

Another aspect we assessed was whether calibrating the software for individual physiognomy features enhanced emotion identification. We found only minor differences between results with and without calibration. Except for expressions of sadness and disgust, the performance of the calibrated analysis is marginally better than without calibration. However, the differences between the two analysis options seem negligible and unsystematic. Compared to the effort necessary for implementing the calibration, the resulting improvements seem minuscule and dispensable, particularly when dealing with large sets of videos.

Compared to emotion classification results obtained with FR 6 [6], version 7 performed somewhat poorer in certain conditions. Firstly, these authors’ mean percentage of correct classification was higher (88%) than that what we achieved in our study (80%). Furthermore, the range of correct classifications across emotion conditions was more favorable in their study with 76% to 94%, compared to 51% to 100% in our study. Only for expressions of happiness and anger did FR7 yield slightly better results than its predecessor. However, the differences between our studies and the one by Lewinski et al [6] may also be due to the different stimulus materials used. Although both studies used high quality frontal recordings of the face, Lewinski et al’.s [6] analysis was based on stills while this study relied on video sequences. Maybe the coding of videos with dynamic expressions such as the SMoFEE dataset is more demanding for FR and thus more prone to errors than coding prototypical stills. Taking into account that the SMoFEE sequences are typical but not completely standardized emotion expressions, comparatively lower detection rates than for fully standardized stills can be expected. Nevertheless, the mean correct identification of 80% of such recordings suggest that FR 7 provides an overall valid classification of emotional expressions. Another reason for the difference between our findings and Lewinski et al’.s [6] may be that the data basis on which FR 7 was trained was larger than the one used for FR 6, and the general face model was improved from FR 6 to FR 7 [8]. More generally, because different software packages and different versions of a given package use different data sets for training their algorithms, some performance differences between different applications of these packages and versions are to be expected, particularly if they are applied to different and novel sets of pictures. It would therefore be helpful for researchers interested in comparing the performance of different versions of software like FR if manufacturers of such software made all technical information about the training, validation, and classification approach of each version of the software permanently available on their website or an open-science resource such as OSF.

### Convergent validity of manual and automated AU coding

To evaluate convergence between manual and automated coding of specific AUs, we focused in our correlation analyses for each AU only on those emotions for which it was a key ingredient and therefore should also show sufficient variation (i.e., correlations with grey background in Table 2). In addition, we proceeded based on the assumption that inter-coder correlation coefficients above .40 indicate fair, and values above .60 good agreement [28]. Taking these considerations into account, across emotional expressions agreement was good for AUs 1 and 2, fair for AUs 9, 17, and 20, mostly fair (i.e., with a minority of coefficients below .40) for AUs 4 and 5, and insufficient for AUs 6, 7, 10, 12, 15, 23, 24, 25, 26, and 27. AUs 14, 18, and 43 were not part of any prototypical emotion expression and interpreting their correlation coefficients may therefore be hampered a lack of variation (i.e., range restriction; see Table 3). Convergence for these latter AUs may be more meaningfully tested in future studies that specifically target expressions involving these AUs.

The low convergence coefficients for AUs 12 (Lip Corner Puller) and 26 (Jaw Drop) pose an unexpected result as the features of these AUs are rather distinctive and deficient detection of these activations thus seems unlikely. The latter conclusion is also underscored by the acceptable levels of between-measure agreement according to the F1 index (Table 4). For AU 12, the result may have been due in part to a ceiling effect for the expression of happiness, limiting the extent to which FACS and FR measurements could covary across their full scales. For AU 26, the difficulty of differentiating between this AU and AUs 25 and 27 might have caused low convergence between manual and automated coding concerning the activated AU. Taken together, 7 of the 17 AUs with meaningful variation (excluding AUs 14, 18, and 43) offer fair to good convergence according to their correlation coefficients (> .40) while the rest falls below this threshold (< .40).

Lewinski et al. [6] report a weighted average agreement index (F1) of .67 for coding AUs with FR 6 and FACS. This is higher than the average F1 value we observed in the present study (0.63). However, their average was based on only 17 AUs assessed by FR version 6, whereas ours includes three additional AUs assessed by FR version 7 (AUs 18, 27, and 43), all of which yielded low agreement between FR and FACS. If these are omitted from averaging, our average agreement index is 0.69 and thus even slightly better than the one reported by Lewinski et al. Similar to these authors, we conclude from our data that FR 7 does a better job detecting basic emotions than measuring individual AUs. Lewinski et al [6] offered two potential reasons for this discrepancy between emotion classification and AU measurement that we think are relevant for interpreting our findings as well. The first is that the number of individual AUs into which an emotional expression can be classified is much higher (20 in our study) than the number of basic emotions (6 in our study) and the likelihood for classification errors increases proportionately. The second is that AU measurement really requires expert knowledge, both from human coders and from software systems, whereas overall emotion classification can be done by naïve human coders as well as less demanding software algorithms. Hence, AU coding is simply harder both for human coders and for software like FR 7.

Another possible cause for the lack of congruence between manual and automated AU codings might have been the differences between each method’s stimulus material. For FACS coding, we extracted and used stills showing a maximum number of maximal AU activations in the videos. This approach is based on the assumption that in a maximally activated emotion expression, all constituting AUs reach their maximum simultaneously in about the same fraction of a second. FR coding of AUs, in contrast, did not rely on this assumption and instead measured AU activation over the entire time course of each dynamically encoded emotion in the videos. The maximum AU levels we obtained from this procedure may not necessarily have emerged simultaneously and may therefore be somewhat at variance with those obtained via FACS. The large discrepancies in the frequency with which some AUs (e.g. 15, 18, 20, 23, 24, and particularly 43) were detected by FACS versus FR (see Table 4) support this argument.

### Implications for applications

What are some of the implications of our findings for using FR 7 (versus FACS) in future research? At first blush, our results for emotion classification suggests that FR 7 is more efficient at classifying and quantifying the intensity of basic emotional expressions than FACS, particularly when it comes to video, as opposed to stills. Hence, FR 7 could be applied to measuring individuals’ emotional expressions in continuously filmed therapy sessions, laboratory interactions, field settings, and so on. However, how well FR 7 will perform will likely depend on the quality of the video material: how well the target person is visible from the front, as opposed to from an angle or moving around, how well the face is lit, whether only one target person is continually visible or other individuals enter and leave the frame, and many other potential distortions. With regard to these factors, our study used material with optimal quality, and the accuracy we achieved both for FR 7 and for FACS therefore probably represents the upper-bound. Although FR 7 is designed to also detect emotions under non-optimal conditions, there is a gradient of accuracy, with the highest levels obtained with material filmed under optimal conditions like in our study and decreasing levels as filming conditions deteriorate.

One possible danger that we were unable to evaluate in our present study but that should be addressed in future work is to what extent FR 7 will be more prone to misclassifying emotional expressions under non-optimal conditions. This is particularly likely for the fear expression, but to a lesser extent also for almost all other expressions except happiness. If left unchecked, such misclassifications could accumulate over the course of a long video, yielding an increasingly invalid aggregate assessment of the emotional expressions of a given target person. On the other hand it is also conceivable that if measurement errors occur due to chance and do not reflect a particular bias to misclassify a given emotion systematically, aggregation across many measurements (e.g., 25 frames per second in film material) will yield a particularly accurate picture of a target person’s emotional dynamics. To resolve these issues, we suggest that more work is needed in which trained actors deliberately encode series of emotional expressions in more natural way and settings and under non-optimal filming conditions and FR 7 and FACS codings of such film clips are then compared to the intended, encoded emotions as well as to each other.

We expect that obtaining high accuracy will be even more daunting for AU codings from non-optimal video material. As our results show, even a relatively straightforward classification of affect into positive and negative responses based on corrugator and zygomatic activations, as proposed by Cacioppo and colleagues (e.g., [34], [35]), can be challenging, as within-emotion convergence between FR and FACS measurements for the corresponding AUs (4, 6, and 12) falls between .08 and .53 (Spearman correlations) even under optimal conditions. However, we cannot rule out that correlation coefficients underestimate the true level of convergence, particularly when the activation of an AU or set of AUs is near a ceiling and correlation coefficients are attenuated due to restricted variances, as in the case of AU 12 in the context of a happy expression. The satisfactory overall level of agreement according to the F1 index for AU 12—and also for AUs 4 and 6 –suggests that convergence assessed via correlation may not tell the whole story under conditions characterized by range restriction.

To ensure accurate classification of AU activations it is of course absolutely critical to capture facial features on video under optimal conditions (i.e., well lit portrait shots with little overall body movement captured on high-resolution HD video). This is something that may be easier to achieve under some circumstances (e.g., therapy sessions, lab tasks) than others (e.g., videos obtained in field settings or with webcams). Nevertheless, the more researchers pay attention to capturing “facial action” of their research participants on video under optimal conditions, the more likely will the codings resulting from FR 7 analysis be accurate and valid. Only then can the greater efficiency of the software approach to measuring facial emotion be exploited to its full extent.

### Limitations

In addition to the limitations we already discussed, the sample of this study was composed of middle aged Caucasians only. Thus the reported validity of the software is restricted to such subjects, as features like age and ethnicity can influence the performance of FR. This study can only be used as an orientation. For other types of sample and video material, the validity of FR 7 codings should be tested in pilot work involving test participants deliberately encoding relevant emotional expressions under typical filming conditions.

Another critical aspect is that neutral expressions were left out in the examination of the emotion classification. This was due to the usage of video sequences containing neutral expression elements and the risk of inflationary categorization of expressions as neutral regardless of the emotion expression as stated in the method section. This leaves a lack of validity indicators for the coding of intentionally neutral expressions as neutral as well as the deficient coding of emotion expressions as neutral. For further analysis of video sequences the neutral expression parts should be extracted from intentional emotion expressions to avoid this problem.

## Conclusion

The central objective of this study was the validation of FR as an economic alternative to manual FACS coding. Automated emotion classification and AU coding was assessed using a dataset of instructed emotional expressions. Adequate validity for the automated method can be reported. We found that emotion classification outperforms the AU coding. Emotion expressions were correctly classified in 80% of the cases whereas the aggregated validity for AU codings was in the medium range.
